# Identification of *FAT4* as a positive prognostic biomarker in DLBCL by comprehensive genomic analysis

**DOI:** 10.1007/s10238-023-01018-z

**Published:** 2023-02-22

**Authors:** Liyang Lv, Xiaolong Qi, Chun Wang, Yutong Ma, Yuling Nie, Renaguli Abulaiti, Fang Zhang, Qiping Shi, Zhen Kou, Muhebaier Abuduer, Shunsheng Zhai, Li An, Qin Huang, Zailinuer Gu, Qiuxiang Ou, Hong Liu, Zengsheng Wang, Yang Shao, Zhenzhu Sun, Ling Fu, Xiaomin Wang, Min Mao, Yan Li

**Affiliations:** 1 Department of Hematology, The People’s Hospital of Xinjiang Uygur Autonomous Region, No. 91, Tianchi Road, Urumqi, 830001 Uygur Autonomous Region China; 2 Department of Pathology, The People’s Hospital of Xinjiang Uygur Autonomous Region, Urumqi, 830001 China; 3 Geneseeq Research Institute, Nanjing Geneseeq Technology Inc., Nanjing, 210000 China; 4 School of Public Health, Nanjing Medical University, Nanjing, 211166 China

**Keywords:** DLBCL, *FAT4*, *TP53*, IPI score, Prognosis

## Abstract

The molecular landscapes of diffuse large B-cell lymphoma (DLBCL) remained to be comprehensively investigated with an urgent need to identify novel prognostic biomarkers guiding prognostic stratification and disease monitoring. Baseline tumor samples of 148 DLBCL patients were analyzed using targeted next-generation sequencing (NGS) for mutational profiling, whose clinical reports were retrospectively reviewed. In this cohort, the subgroup of old DLBCL patients (age at diagnosis > 60, *N* = 80) exhibited significantly higher Eastern Cooperative Oncology Group scores and International Prognostic Index than their young counterparts (age at diagnosis ≤ 60, *N* = 68). As revealed by the NGS results, *PIM1* (43.9%), *KMT2D* (31.8%), *MYD88* (29.7%), and *CD79B* (27.0%) were identified as the most frequently mutated genes. Aberrations of genes of the immune escape pathway were significantly enriched in the young subgroup, while the altered epigenetic regulators were more abundant in the old patients. *FAT4* mutation was identified as a positive prognostic biomarker, associated with longer progression-free survival and overall survival in the entire cohort and the old subgroup, using the Cox regression analyses. However, the prognostic function of *FAT4* was not reproduced in the young subgroup. We comprehensively analyzed the pathological and molecular characteristics of old and young DLBCL patients and demonstrated the prognostic value of *FAT4* mutation, which requires further validation with sizable cohorts in future research.

**Supplementary Information:**

The online version contains supplementary material available at 10.1007/s10238-023-01018-z.

## Introduction

Diffuse large B-cell lymphoma (DLBCL) is the most common (40%) and an aggressive form of non-Hodgkin lymphoma [1, 2]. DLBCL occurs at any age, but it is more common in the elder, with a median age at diagnosis of 66 years and one-third of patients are over 75 years [3]. Previous studies have shown distinct clinical and genetic characteristics between the young (≤ 60) and old (> 60) DLBCL patients [3]. For instance, old DLBCL patients are usually associated with poor prognostic factors such as late Ann Arbor stage, high-level lactate dehydrogenase (LDH) in serum, multiple extranodal involvements, non-germinal center B‐cell (non-GCB) phenotype, *MYC/BCL2* double expression, and Epstein-Barr virus (EBV) infection [3, 4].

The treatment strategy for old DLBCL patients is also different from young patients as they usually exhibit poorer health conditions and intolerance to immunochemotherapy. Thus, personalized treatment based on unique molecular features is becoming attractive. With the broad application of next-generation sequencing (NGS) in clinical practice, mutational landscape and signaling pathway studies in DLBCL have provided novel insights into pathogenesis. However, the genome of DLBCL presented a high degree of complexity with great variability of gene alterations across individual cases (ranging from 0 to 92 alterations) [5]. As reported, old DLBCL patients tended to accumulate genomic alterations, such as higher mutational frequencies of *MYD88*, *PIM1*, and *CD79B*, and changes in tumor immune microenvironment [3, 4, 6]. In addition, whole-exon sequencing identified a number of recurrent mutations, both canonical and not previously identified ones, which were involved in some tumorigenesis- and treatment-related signaling pathways [7, 8]. Nevertheless, studies on the prognosis of DLBCL patients, especially for the elder, and its correlation with gene-level or pathway-level mutational features are limited. Herein, we, respectively, reviewed the clinical and pathological reports of 148 DLBCL patients whose baseline tumor tissue underwent targeted NGS covering 475 lymphoma-related genes. In this study, we comprehensively compared the mutational landscape of young and old DLBCL subgroups and identified a novel prognostic factor, *FAT4*, especially in the old subgroup.

## Methods

### Patient enrollment

A total of 148 DLBCL patients primarily diagnosed between March 2009 and March 2021 at People’s Hospital of Xinjiang Uygur Autonomous Region were enrolled in this study following the guideline of WHO Classification of Tumours of Haematopoietic and Lymphoid Tissues [9]. Patients without complete clinical data or lost to follow-up were excluded. Other exclusion criteria included no medical treatment, radio-/chemotherapy prior to enrollment, complications of other hematologic neoplasms, or malignant wasting diseases. The clinical and pathological information at diagnosis were collected and reviewed, including age, sex, serum LDH level, Ann Arbor stage, primary site, number of extranodal involvement, Eastern Cooperative Oncology Group (ECOG) performance status, CD5 expression, MYC/BCL2 double expression, and cell of origin (COO). The International Prognostic Index (IPI) score was evaluated in all patients who were assigned one point for each negative prognostic factor (age at diagnosis over 60 years, upper limit of normal serum LDH level, Ann Arbor stage III/IV, ECOG performance status ≥ 2, and extranodal involvement > 1 site) [10]. This study was approved by the ethics committee of the People’s Hospital of Xinjiang Uygur Autonomous Region (Approval No. KY2019101001). All patients provided written informed consent to participate in the study and provide samples for tumor genetic profiling.

### Immunohistochemistry (IHC)

IHC was performed on 4 µm Formalin-Fixed Paraffin-Embedded (FFPE) tissue samples using Bond-Max Automated IHC Stainer (Leica Biosystems Inc., Wetzlar, Germany). The monoclonal antibodies against human CD5 (clone SP19, Zymed, TX, USA), CD10 (clone SP67, Novocastra, TX, USA), BCL2 (clone EP36, Novocastra, TX, USA), BCL6 (clone PF16 + PG + B6p, Novocastra, TX, USA), MUM1 (clone MUM1P, Dako, TX, USA), and MYC (clone Y69, Novocastra, TX, USA) were used as primary antibodies. The DLBCL subtypes of GCB or non-GCB were categorized according to the Hans algorithm based on CD10, BCL6, and MUM1 expression [11]. IHC stains were independently scored by two pathologists (Chun Wang and Zhenzhu Sun). Cases were identified as positive if more than 30% of tumor cells were stained with a specific antibody in five randomly selected high-quality staining fields under 400× magnification. MYC/BCL2 double expression was defined by MYC and BCL-2 with cutoff values of 40% and 50%, respectively [12].

### Treatment and response evaluation

All patients received standard R-CHOP (rituximab, cyclophosphamide, doxorubicin, vincristine, and prednisone; R-CHOP), R-mini-CHOP (dose-reduced R-CHOP), or R-COP (standard R-CHOP without doxorubicin) for four to six cycles according to their age and physical conditions, followed by two cycles of rituximab monotherapy. Twenty-three of them also received autologous stem cell transplantation (ASCT) for consolidation therapy. The second-line treatment included DHAP (dexamethasone, cytarabine, and platinum), ICE (ifosfamide, carboplatin, and etoposide), and GDP (gemcitabine, dexamethasone, and cisplatin). The response to treatment was evaluated based on imagological examinations (CT, MRI, or PET/CT) [13]. Overall survival (OS) was calculated as the duration from the date of DLBCL diagnosis to the date of death of any causes or the last follow-up date (December 2021). Progression-free survival (PFS) was defined as the period from the date of diagnosis to the date of progression, recurrence, or last follow-up. The median follow-up period length was 29 months (range: 2–144 months).

### Next-generation DNA sequencing and analysis

The genomic DNA was extracted from formalin-fixed, paraffin-embedded (FFPE) tissue samples whose tumor cell content was over 20%, using the QIAamp DNA FFPE Tissue Kit (Qiagen, Hilden, Germany) and following the manufacturer’s protocol. The extracted DNA was then quantified using the dsDNA HS Assay Kit on a Qubit 2.0 Fluorometer (Life Technologies, Darmstadt, Germany).

Targeted NGS was performed using a panel (Hemasalus™) of exons and splice sites of 475 genes that are recurrently mutated in B-cell lymphomas [14]. NGS was performed at a Clinical Laboratory Improvement Amendments (CLIA) and College of American Pathologists (CAP)-accredited testing laboratory (Nanjing Geneseeq Technology, Inc, Nanjing, China). Sequencing libraries were prepared using the KAPA Hyper Prep Kit (KAPA Biosystems) and sequenced on a HiSeq 4000 NGS platform (Illumina) [15]. Sequencing data were processed as previously described [16]. In brief, the data were first demultiplexed and the FASTQ file was subjected to quality control to remove low-quality data or N bases. Qualified reads were mapped to the reference human genome, hg19, using the Burrows-Wheeler Aligner. The Genome Analysis Toolkit (GATK 3.4.0) was used to perform local realignment around indels and base quality score recalibration. Picard was used to remove PCR duplicates. VarScan2 was used for the detection of single-nucleotide variants and insertion/deletion mutations. A mutant allele frequency cutoff of 0.5% was used for tissue samples. ADTEx was used to identify copy number variations. A cutoff log2 ratio was set at ± 0.6 for copy number changes (corresponding to a 1.5-fold copy number gain and 0.65-fold copy number loss).

### Statistics

Data were analyzed using R 3.6.3. Categorical variables between groups were compared using χ2 or Fisher’s exact test. Kaplan–Meier method was used to determine median PFS/OS and the significance of survival analysis was determined by the log-rank test. Univariate and multivariate Cox regression analysis was used to identify prognostic factors. A *p *value < 0.05 was defined as statistically significant.

## Results

### Overview of DLBCL patients

As shown in Table 1, a total of 148 DLBCL patients were enrolled in this study with a median age at diagnosis of 62 years (range: 23–93 years) and an equal proportion of male and female (49.3% vs. 50.7%). IPI scores were evaluated based on clinical and pathological features as described in Methods and over half (82/148, 55.4%) of the patients had high/intermediate high (≥ 3) IPI scores. In addition, GCB and non-GCB subtypes each accounted for approximately half of the cohort (51.4% vs. 48.6%). All patients received standard/dose-reduced R-CHOP or R-COP as the first-line treatments. Notably, the ASCT procedure was performed in 23 patients after 1st-line treatment and less than one-quarter of patients (33/148) received 2nd-line therapies.

**Table 1:** The clinical and pathological characteristics of patients

Characteristics	Patients (*N* = 148)
Age	
Median (range)	62 (23–93)
Sex	
Male	73 (49.3%)
Female	75 (50.7%)
LDH	
Normal	80 (54.1%)
High	68 (45.9%)
ECOG	
< 2	61 (41.2%)
≥ 2	87 (58.8%)
Ann Arbor Stage	
I/II	38 (25.7%)
III/IV	110 (74.3%)
Primary Site	
Intranodal	81 (54.7%)
Extranodal	67 (45.3%)
Number of Extranodal tumors	
< 2	108 (73.0%)
≥ 2	40 (27.0%)
IPI score	
< 3	66 (44.6%)
≥ 3	82 (55.4%)
Cell of Origin	
GCB	76 (51.4%)
Non-GCB	72 (48.6%)
CD5 Expression	
Negative	140 (94.6%)
Positive	8 (5.4%)
MYC/BCL2 double expression	
Negative	122 (82.4%)
Positive	26 (17.6%)
ASCT	
No	125 (84.5%)
Yes	23 (15.5%)
Line of Treatment	
1st-line only	115 (77.7%)
1st/2nd-line	33 (22.3%)

*LDH* lactate dehydrogenase, *ECOG* Eastern Cooperative Oncology Group, *IPI* International Prognostic Index, *AST* autologous stem cell transplantation, *GCB* germinal center B‐cell

A total of 80 patients whose age at diagnosis was over 60 years were allocated to the old subgroup, while the rest 68 patients were classified into the young subgroup (Table S1). Patients in the old subgroup were significantly associated with intermediate high/high (≥ 3) IPI scores (*p* < 0.00001) and high ECOG status (≥ 2, *p* < 0.001). The MYC/BCL2 double expression was also more enriched in the old subgroup (23.8% vs. 10.3%, *p* < 0.05), whereas no significant differences were found between the old and young subgroups in serum LDH level, Ann Arbor stage, primary tumor site, the number of extranodal tumors, and COO.

### Mutational landscape of old and young DLBCL patients

**Fig. 1:**
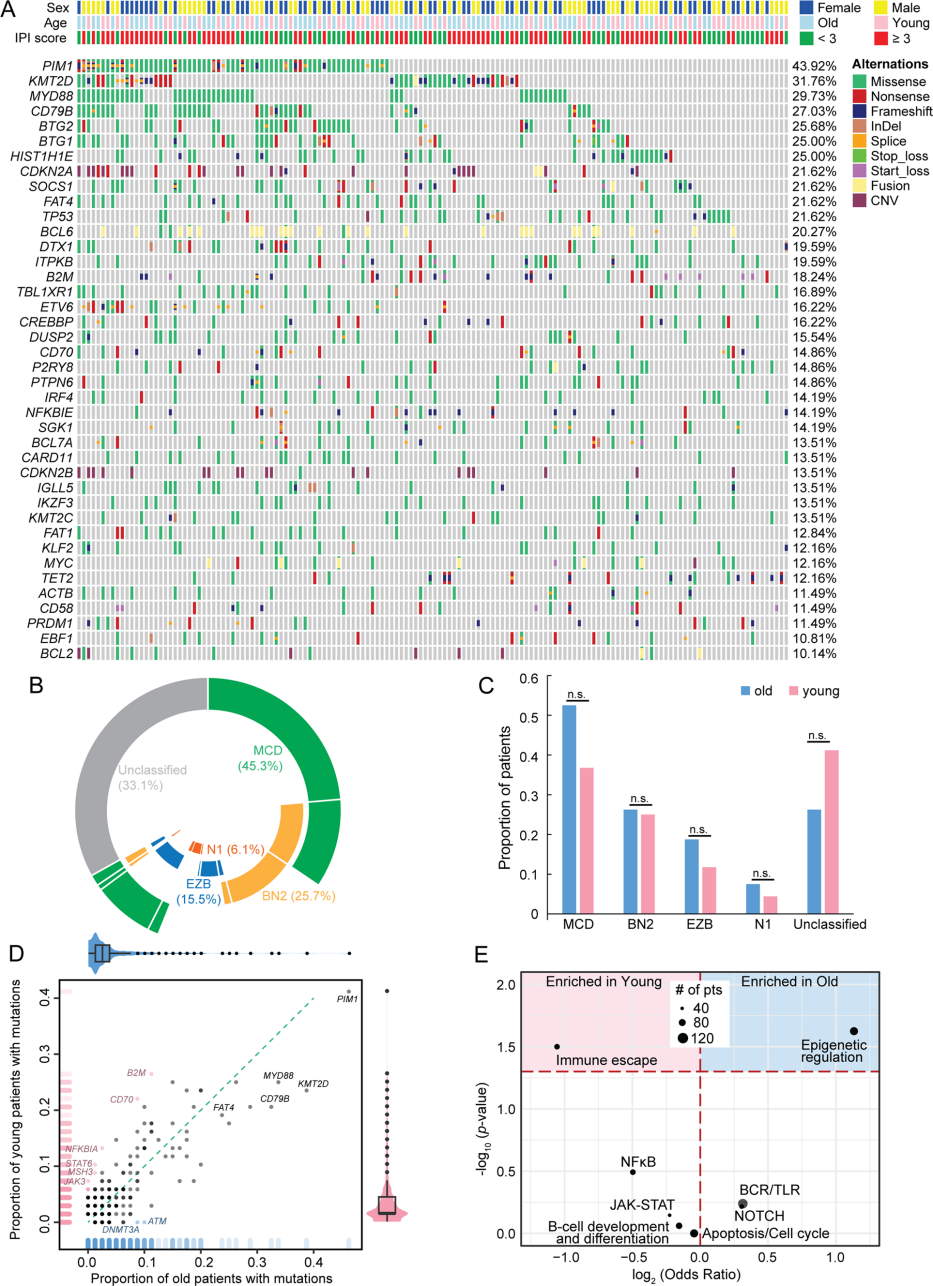
Mutational landscape and genetic subtype distribution of DLBCL. **A** Somatic mutations, structural variants (fusions), and copy number variants detected by the hybrid capture-based NGS are shown by the oncoprint plot. Clinical features and alteration subtypes are colored as the legend. **B** The proportions of four genetic subtypes, including MCD (based on MYD88 and CD79B), BN2 (based on BCL6 and NOTCH2), EZB (based on EZH2 and BCL2), and N1 (based on NOTCH1), are shown by the four layers of the pie chart, respectively. **C** Proportion of patients with each genetic subtypes or unclassified are shown and *p* values are calculated using Fisher’s exact tests. **D** The x- and y-axis show the proportion of old and young patients with mutations of each gene. The dots colored in blue or pink represent the genes that are significantly enriched in the old or young subgroup, respectively. The violin plots and the gradient-colored perpendicular markers on the x- and y-axis demonstrate the distribution of mutational frequencies of all detected genes in the old and young subgroups. The green dashed line represents the equal distribution in the old and young subgroups. **E** Eight DLBCL-related pathways are analyzed using fisher’s exact test to compare their frequencies in the old and young subgroups. The area above the red dashed line is statistically significant representing the enrichments in the young (left, pink) and old (right, blue), respectively

The FFPE tumor samples collected at baseline were subjected to targeted NGS covering 475 genes that are related to B-cell lymphomas. We defined the genes whose mutational frequency was over 10% in this cohort as “high-frequency,” which were shown in Fig. 1A. *PIM1* (43.9%) and *KMT2D* (31.8%) were the two most frequently mutated genes followed by *MYD88* (29.7%) and *CD79B* (27.0%). As *MYD88* and *CD79B* mutations were the markers of the MCD subtype, one of the four prominent genetic subtypes in DLBCL [17], nearly half (67/148) of the cohort, were classified into the MCD subtype (Fig. 1B). BN2 was the second dominant (25.7%) genetic subtype which was identified based on *BCL6* fusions and *NOTCH2* mutations. EZB (based on *EZH2* and *BCL2* mutations) and N1 (based on *NOTCH1* mutation) subtypes were relatively infrequent in this cohort (15.5% and 6.1%, respectively). Approximately one-third (49/148) of patients could not be classified into any genetic subtypes. By comparing the old and young subgroups, we found that 26.3% of old patients were not classified into any of the four genetic subtypes (MCD, BN2, EZB, and N1), which was slightly lower than that in the young subgroup (41.2%, Fig. 1C), and no significant enrichments of the four genetic subtypes were observed between the old and young subgroups.

To further investigate the mutational landscape of old and young DLBCL patients, we compared the mutational frequencies of all detected genes in the old and young subgroups (Fig. 1D). In both old and young patients, most mutated genes were detected in less than 10% of patients. Old patients tended to harbor more top mutated genes such as *PIM1*, *KMT2D*, *MYD88*, and *CD79B* but not statistically significant. *ATM* (10% vs. 0%, *p* = 0.008) and *DNMT3A* (8.75% vs. 0%, *p* = 0.016) mutations were exclusively detected in old patients. In contrast, *B2M* (11.25% vs. 26.47%, *p* = 0.020), *CD70* (8.75% vs. 22.06%, *p* = 0.035), *NFKBIA* (2.5% vs. 13.2%, *p* = 0.024), and *STAT6* (1.25% vs. 10.3%, *p* = 0.024) were significantly enriched in the young subgroup.

We next compared the alteration frequencies at the pathway level [18] between the old and young subgroups and found that the immune escape pathway was significantly enriched in the young subgroup (52.9% vs. 35%, *p* = 0.032), which contained two young-enriched genes, *B2M* and *CD70* (Fig. 1E). In addition, the proportion of patients harboring epigenetic regulation-related gene mutations was significantly higher in the old subgroup (71.3% vs. 52.9%, *p* = 0.03). No enrichment preferences were demonstrated in other pathways listed in Table S2.

### *FAT4* mutation is a good prognostic factor for DLBCL

Up to December 2021, the median follow-up period length of this cohort was 29 months (range: 2 to 144 months). The median PFS was 75 months, and the median OS was not reached yet. The prognosis of patients with high/intermediate high IPI scores (≥ 3) was dramatically poorer than those with IPI scores < 3 (Figure S1A-B, log-rank *p* < 0.001). As the age at diagnosis is one of the scoring standards for IPI score, the PFS and OS of old patients were relatively shorter than young patients, but it was not an independent prognostic marker (Figure S1C-D). To explore the potential genetic prognostic biomarkers in DLBCL, we first performed the univariate analysis for PFS based on the Cox regression model with the baseline clinical features and 12 top frequently mutated genes, which were all detected in over 20% of patients. As shown in Table 2, among the analyzed features, COO, Ann Arbor stage, IPI scores (*p* < 0.05), as well as the mutations of *TP53* and *FAT4* (adjusted *p* < 0.1), were significantly associated with PFS. As the Ann Arbor stage was one of the scoring factors for IPI scores, it was excluded from the multivariate analysis, and the rest four features, including COO, IPI scores, *TP53* mutations, and *FAT4* mutations, remained as independent and significant prognostic factors for predicting PFS.

**Table 2:** Univariate and multivariate analysis of PFS based on Cox regression model with clinical features and top mutated genes

	Factor		Univariate			Multivariate	
			HR (95% CI)	*p *value	Adjusted *p* (FDR)	HR (95% CI)	*p *value
Clinical feature at baseline	Age	Old versus young	1.33 (0.81, 2.20)	0.263	–	–	–
	Sex	Female versus Male	0.66 (0.40, 1.08)	0.100	–	–	–
	Cell of origin	Non-GCB versus GCB	1.67 (1.02, 2.75)	0.043	–	1.76 (1.06, 2.92)	0.03
	Ann Arbor stage	III/IV versus I/II	3.21 (1.53, 6.75)	0.002	–	–	–
	LDH level	Elevated versus normal	1.41 (0.86, 2.31)	0.169	–	–	–
	Primary site	Extranodal versus intranodal	0.70 (0.42, 1.16)	0.164	–	–	–
	CD5 expression	Positive versus negative	0.46 (0.11, 1.86)	0.273	–	–	–
	MYC/BCL2 double expression	Positive versus negative	0.63 (0.29, 1.40)	0.259	–	–	–
	Number of extranodal tumors	≥ 2 versus 0–1	0.89 (0.51, 1.55)	0.683	–	–	–
	ECOG	≥ 2 versus 0–1	1.50 (0.89, 2.52)	0.125	–	–	–
	IPI score	≥ 3 versus ≤ 2	2.08 (1.22, 3.56)	0.008	–	2.93 (1.67, 5.14)	< 0.001
Gene Mutation	*PIM1*	Mutation versus wildtype	0.70 (0.42, 1.16)	0.171	0.276	–	–
	*KMT2D*		1.43 (0.87, 2.37)	0.162	0.276	–	–
	*MYD88*		0.83 (0.48, 1.43)	0.493	0.592	–	–
	*CD79B*		1.07 (0.62, 1.85)	0.798	0.870	–	–
	*BTG2*		0.52 (0.27, 0.99)	0.047	0.141	–	–
	*BTG1*		0.65 (0.35, 1.22)	0.184	0.276	–	–
	*HIST1H1E*		0.62 (0.28, 1.37)	0.240	0.319	–	–
	*CDKN2A*		1.01 (0.56, 1.80)	0.988	0.988	–	–
	*FAT4*		0.35 (0.16, 0.77)	0.009	0.054	0.28 (0.13, 0.63)	0.002
	*SOCS1*		0.55 (0.28, 1.08)	0.083	0.199	–	–
	*TP53*		2.33 (1.36, 3.97)	0.002	0.024	3.06 (1.74, 5.38)	< 0.001
	*BCL6*		0.45 (0.21, 0.94)	0.034	0.135	–	–

**Fig. 2:**
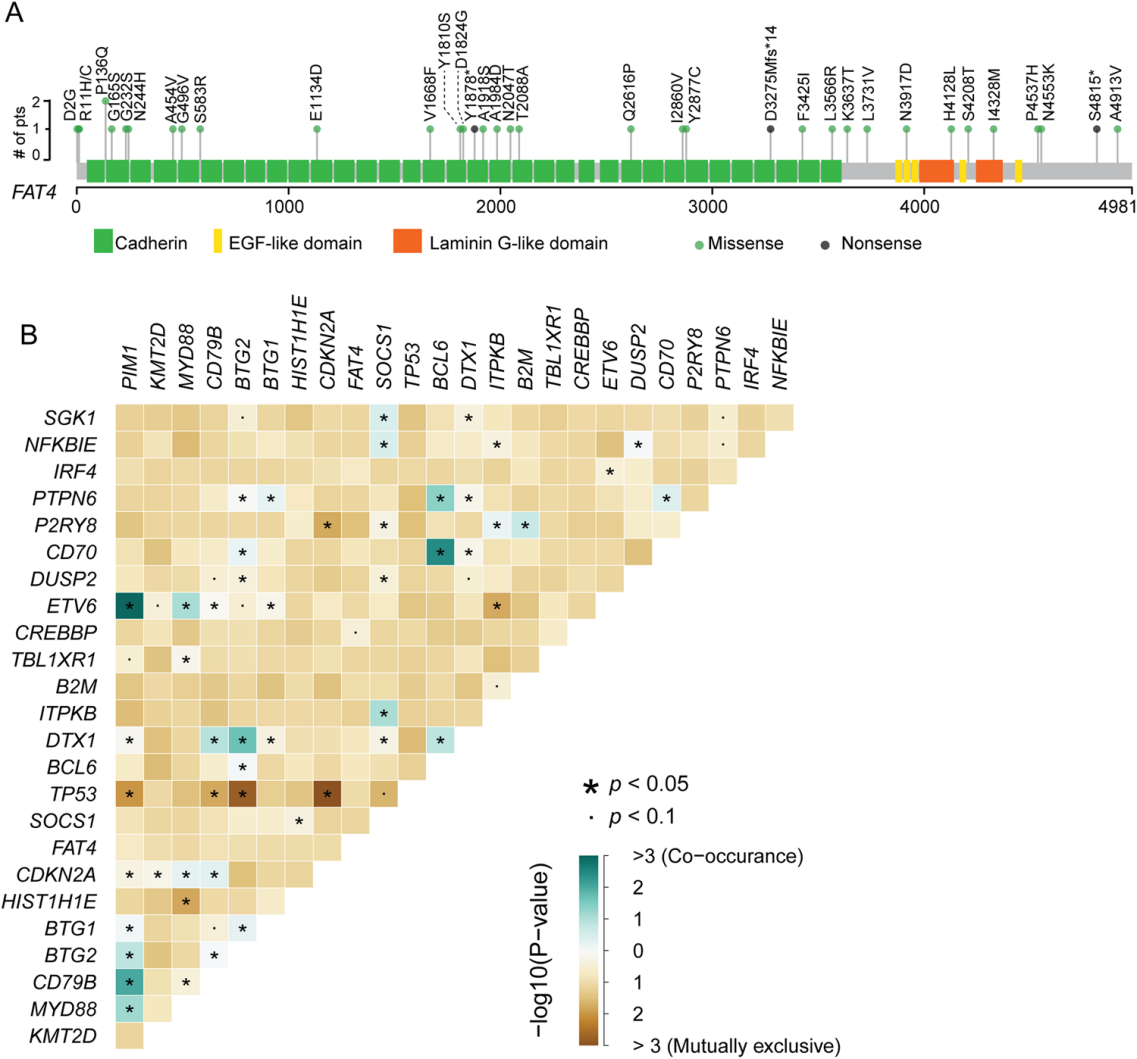
*FAT4* mutation spectrum and mutational exclusive analysis. **A** Structure of *FAT4* gene is shown with different colors corresponding to domains. Each circle represents one patient with green representing missense mutations and gray representing nonsense mutations. **B** Heatmap of mutually exclusive or co-occurring 25 top altered genes including mutations, copy number variants, and fusions

*FAT4* is a member of the FAT family which encode large transmembrane proteins with Cadherin repeats, epidermal growth factor (EGF)-like domains, and Laminin G-like domains (Fig. 2A). The majority of *FAT4* mutations detected in this cohort were missense mutations that spanned the whole protein structure without any mutation hot spots. Only one recurrent mutation, P136Q, was detected among all *FAT4* mutations. We then investigated the mutational exclusiveness between *FAT4* and other frequently mutated genes. As shown in Fig. 2B, *FAT4* mutation was not significantly mutually exclusive or co-occurred with other gene mutations, except for a trend of exclusiveness with *CREBBP* (*p* < 0.1). However, *TP53* mutations were mutually exclusive with *PIM1*, *CD79B*, *BTG2*, and *CDKN2A* alterations (*p* < 0.05) and *PIM1* was frequently co-mutated with *MYD88*, *CD79B*, *BTG1/2*, and *ETV6* (*p* < 0.05)*.*

**Fig. 3:**
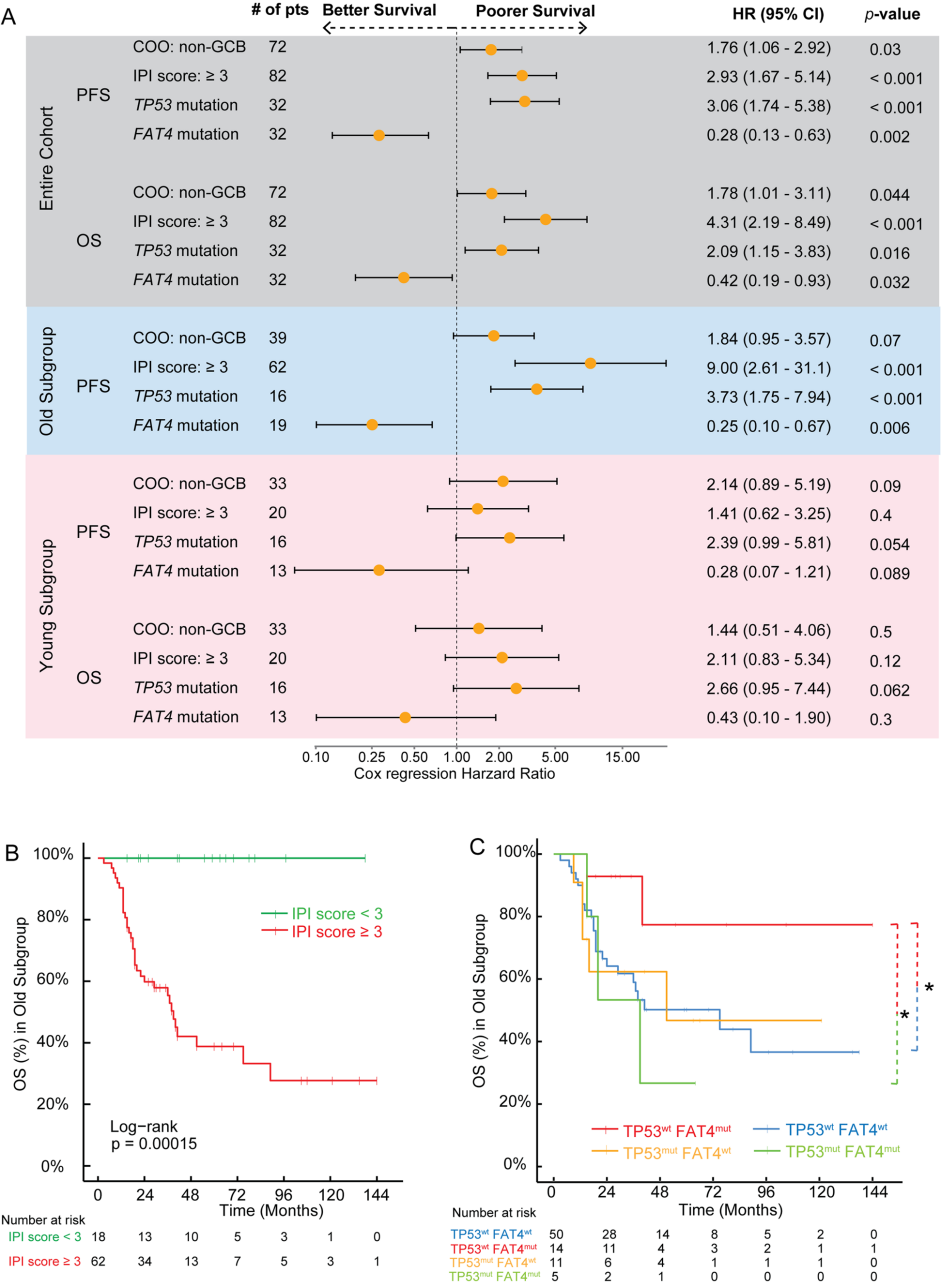
High/intermediate high IPI score, *TP53,* and *FAT4* mutations are independent prognostic factors. **A** The forest plots of PFS/OS of the entire cohort and PFS of old patients are generated based on multivariate Cox regression analysis with IPI score (high/intermediate high IPI ≥ 3 vs. low/intermediate low IPI < 3), *TP53* (mutation vs. wild-type), and *FAT4* (mutation vs. wild-type). **B**–**C** The KM survival curves of OS in the old subgroup were classified based on IPI score and the mutational status of *TP53* and *FAT4*

As shown in Fig. 3A, the four prognostic factors identified from the PFS analysis remained independent when predicting OS in this cohort. Then, we investigated their prognostic role in the old and young subgroups, respectively. In the old subgroup, IPI score, *TP53,* and *FAT4* mutations were still the independent prognostic factors for PFS based on the multivariate Cox regression model (Fig. 3A). As no death event occurred in the old patients with low/intermediate low (< 3) IPI scores, the Cox regression model was not applicable for OS analysis, but high/intermediate high (≥ 3) IPI score was significantly associated with inferior OS in the old subgroup (log-rank *p* < 0.001, Fig. 3B). Furthermore, *TP53-*wildtype (wt) old patients carrying *FAT4* mutations (*TP53*^wt^*/FAT4*^mut^) showed the best survival outcomes, particularly, the OS of *TP53*^wt^*/FAT4*^mut^ was significantly longer than that of *TP53/FAT4* double-wt or double-mutant old patients (Fig. 3C). Thus, *FAT4* was a novel prognostic factor for both PFS and OS in old DLBCL patients. However, *FAT4* mutation was no longer associated with better PFS or OS in the young subgroup (Fig. 3A and Figure S1 E–F), suggesting the existence of differences in genetic characteristics or carcinogenesis between old and young DLBCL patients.

### Prognostic value of key genes of the JAK-STAT pathway besides *FAT4* in DLBCL

*FAT4* was reported to be closely related to the JAK-STAT pathway, the key players of which were *SOCS1* and *STAT6 *[19]. Previous studies showed that *SOCS1* mutation was a significant predictor of good survival in DLBCL [20, 21] and we also observed a trend of better survival with *SOCS1* mutations (PFS: HR 0.55 [95% CI: 0.28–1.08], *p* = 0.08; Table 2) with a mutational frequency of 21.6% in our cohort. Thus, when combining *FAT4*, *SOCS1*, and *STAT6* together as the representatives of the JAK-STAT pathway, we found that the altered JAK-STAT pathway was strongly associated with longer PFS in the entire cohort as well as in the old and young subgroups, respectively (Figure S2), while the prognostic impact on OS of JAK-STAT pathway alterations was significant in the entire cohort (*p* = 0.007) and the old subgroup (*p* = 0.03) but not in the young subgroup (*p* = 0.15).

## Discussion

In this study, we comprehensively investigated the genetic landscape of DLBCL patients and compared the differences in clinical and molecular characteristics between old and young patients. We observed poorer ECOG performance status, higher IPI scores, and a higher percentage of MYC/BCL2 double expression subtype in the old subgroup but not other clinical and pathological features, such as advanced disease stage, elevated LDH level, and multiple extranodal involvements, which were reported to be more frequently presented in the old DLBCL patients in a previous study [4]. The mutational landscapes of old and young DLBCL patients were semblable, both of which contained a large number of low-frequency gene mutations (< 10% of patients).

We observed a significant enrichment of epigenetic regulation pathway alterations in the old DLBCL patients, involving the mutations of *KMT2D*, *CREBBP*, *EP300* et al. Similarly, Zhu et al. reported a strong correlation between histone acetylation-related gene mutations and age at diagnosis [4]. It is well accepted that epigenetic dysfunction could induce lymphomagenesis and was linked to dismal survival [22, 23]. For instance, mutations of *KMT2D*, *CREBBP*, and *EP300* have been identified as poor prognostic biomarkers in previous studies [24, 25]. Thus, our findings once again supported the critical role of epigenetic regulation, which promoted the implementation of precision epigenetic therapies, especially for old DLBCL patients.

Escaping immune surveillance is a critical prerequisite for tumor progression in many cancer types including DLBCL, either through “hiding” or “defending” themselves [26]. Multiple genetic mechanisms of immune escape have been studied in DLBCL, such as the loss or downregulation of antigen expression and immunosuppressive microenvironment. [27, 28] Here, we observed an enrichment of immune escape pathway alterations in the young subgroup, mainly resulting from the higher frequencies of *B2M* and *CD70* mutations. Jiang et al. reported an increasing number of *B2M* mutations in refractory or relapse DLBCL using deep sequencing, revealing the novel clonal evolution and mutational patterns [29], while *CD70* mutations were reported to be commonly associated with the B2N subtype in DLBCL [17]. The findings in immune modulation largely promoted the development of immunotherapy and guided personalized treatments in DLBCL patients.

*TP53* mutation has been repeatedly proven as a poor prognostic indicator and associated with disease progression in DLBCL [30–32]. Once again, in this study, patients carrying *TP53* mutations exhibited significantly longer PFS and OS than *TP53*^wt^ patients. Strikingly, we found a novel prognostic biomarker, *FAT4*, especially for old DLBCL patients. *FAT4* encodes a cadherin-related protein in the FAT family playing the role of tumor suppressor through Hippo and Wnt/β-catenin signaling pathways, recurrent mutations of which were reported in multiple cancer types, such as gastric cancer [33], myeloma [34], and endometrial cancer [35]. High *FAT4* expression was associated with a favorable prognosis in colorectal cancer [36] and gastric cancer [37]. In addition, Zhuang et al. reported that *FAT4* mutations significantly down-regulated their RNA expression levels and were remarkably enriched in early-stage (I/II) colorectal cancer patients, portending a low recurrence rate and longer PFS [38]. Those prior results of *FAT4* indicated its critical role in tumorigenesis in diverse cancer types but the detailed mechanism remained unclear and requires to be further investigated. Notably, the prognostic impact of *FAT4* has never been reported in hematologic tumors but it was recurrently mutated in primary central nervous system lymphoma [39], gastrointestinal DLBCL [40], and splenic marginal zone lymphoma [41]. Thus, we demonstrated *FAT4* mutation as a favorable prognostic biomarker in DLBCL for the first time, especially for old patients. In our cohort, the mutational frequency of *FAT4* was 23.8% (19/80) in the old subgroup and only five *FAT4-*mutated old patients progressed within the follow-up period. More importantly, three of them concurrently carried *TP53* mutations, a canonical inferior prognostic biomarker. The close relationship between *FAT4* and the JAK-STAT signaling pathway has been well established [19], and *SOCS1*, a known key player of JAK-STAT pathway, was previously identified as a favorable prognostic biomarker in DLBCL [20, 21]. Therefore, the prognostic impact of *FAT4* in DLBCL is consistent with those results of *SOCS1* and the JAK-STAT pathway. Together, our findings suggested FAT4 as a novel prognostic biomarker of DLBCL that requires further investigation in both pre-clinical and clinical settings.

The limitations of this study mainly resulted from the nature of the retrospective study such as restrictive cohort size and non-uniformed therapies, which might lead to insignificant differences in PFS and OS between old and young subgroups. Due to the lack of a public DLBCL cohort with survival outcomes in the same setting as our study, the prognostic function of *FAT* was not validated in the external dataset. Thus, further studies with a larger cohort size are warranted to validate the results reported here and investigate the molecular mechanism of *FAT4* associated with prognosis.

## Conclusions

In conclusion, we comprehensively analyzed the genetic alterations using large-panel NGS and identified a novel favorable prognostic biomarker, *FAT4*, for the first time in DLBCL. The more significant effects of *FAT4* in the old subgroup and *TP53*^wt^ patients indicated the uncovered and complicated molecular mechanisms behind our findings which required prospective studies with sizable cohorts in the future. Our study not only inspired precision medicine in DLBCL but also promoted the application of NGS in DLBCL management, as well as prognostic stratification.

### Supplementary Information

Below is the link to the electronic supplementary material.

Supplementary file1 (PDF 524 kb)

## Data Availability

All data generated or analyzed during this study are included in this published article and its supplementary information files. The datasets used and/or analyzed in the current study are available from the corresponding author upon reasonable request.

## Abbreviations

DLBCL: Diffuse large B-cell lymphoma
LDH: Lactate dehydrogenase
GCB: Germinal center B‐cell
EBV: Epstein–Barr virus
NGS: Next-generation sequencing
ECOG: Eastern Cooperative Oncology Group
COO: Cell of origin
IPI: International Prognostic Index
IHC: Immunohistochemistry
FFPE: Formalin-fixed paraffin-embedded
ASCT: Autologous stem cell transplantation
OS: Overall survival
PFS: Progression-free survival
wt: Wildtype
mut: Mutation
