# Supplementary material for: Identification of FAT4 as a positive prognostic biomarker in DLBCL by comprehensive genomic analysis
Source: Clin Exp Med. 2023 Feb 22;23(6):2675–85. doi: 10.1007/s10238-023-01018-z (PMC10543145; doi:10.1007/s10238-023-01018-z)
Supplement: Supplementary file 1 — Supplementary file1 (PDF 524 kb) [file 10238_2023_1018_MOESM1_ESM.pdf]

Supplementary File

Identification of *FAT4* as a positive prognostic biomarker in DLBCL by comprehensive genomic analysis

Supplementary Table S1. Comparison of the clinical and pathological characteristics between the old and young subgroups

Supplementary Table S2. Pathway enrichment analysis between old and young patients

Supplementary Figure S1. The prognostic impact of IPI score, age, and *FAT4* mutation

Supplementary Figure S2. JAK-STAT pathway alterations are associated with better survival.

Supplementary Table S1. Comparison of the clinical and pathological characteristics between the old and young subgroups

Characteristics		Old subgroup (N = 80)	Young subgroup (N = 68)	*p*-value
Age	Median (range)	71 (61 - 93)	48 (23 - 60)	-
Sex				1
	Male	39 (48.75%)	34 (50.00%)	
	Female	41 (51.25%)	34 (50.00%)	
LDH				0.62
	Normal	45 (56.25%)	35 (51.47%)	
	High	35 (43.75%)	33 (48.53%)	
ECOG				
	< 2	21 (26.25%)	40 (58.82%)	< 0.001
	≥ 2	59 (73.75%)	28 (41.18%)	
Ann Arbor Stage				0.85
	I/II	20 (25.00%)	18 (26.47%)	
	III/IV	60 (75.00%)	50 (73.53%)	
Primary Site				0.74
	Intranodal	45 (56.25%)	36 (52.94%)	
	Extranodal	35 (43.75%)	32 (47.06%)	
Number of Extranodal tumors				0.89
	< 2	58 (72.50%)	50 (73.53%)	
	≥ 2	22 (27.50%)	18 (26.47%)	
IPI score				< 0.00001
	< 3	18 (22.50%)	48 (70.59%)	
	≥ 3	62 (77.50%)	20 (29.41%)	
Cell of Origin				0.98
	GCB	41 (51.25%)	35 (51.47%)	
	non-GCB	39 (48.75%)	33 (48.53%)	
CD5 Expression				0.73
	Negative	75 (93.75%)	65 (95.59%)	
	Positive	5 (6.25%)	3 (4.41%)	
MYC/BCL2 double expression				< 0.05
	Negative	61 (76.25%)	61 (89.71%)	
	Positive	19 (23.75%)	7 (10.29%)	
ASCT				
	Yes	80 (100%)	45 (66.2%)	< 0.00001
	No	0 (0%)	23 (33.8%)	
Line of Treatment				
	1st-line only	60 (75%)	55 (80.9%)	0.43
	1st/2nd-line	20 (25%)	13 (19.1%)	

Note: LDH, lactate dehydrogenase; ECOG, Eastern Cooperative Oncology Group; IPI, International Prognostic Index; AST, autologous stem cell transplantation; GCB, germinal center B-cell.

Supplementary Table S2. Pathway enrichment analysis between old and young patients

Pathway	Gene	All patients N (%)	Old subgroup N (%)	Young subgroup N (%)	*p*-value
NF-kappa B	*TNFAIP3, TBL1XR1, KLHL6, NFKBIE, NFKBIA, REL*	65 (43.9%)	32 (40.0%)	33 (48.5%)	0.32
BCR/TLR	*MYD88, CD79B, CARD11, PRKCB, PTPN6, LYN, GRB2, TLR2, CD79A, SYK, BTK, PLCG2, ITPKB, MALT1, BCL10*	110 (74.3%)	61 (76.3%)	49 (72.1%)	0.58
NOTCH	*NOTCH1, NOTCH2, DTX1, SPEN*	54 (36.5%)	31 (38.8%)	23 (33.8%)	0.61
Epigenetic regulation	*KMT2D, HIST1H1E, CREBBP, EZH2, EP300, KMT2C*	93 (62.8%)	57 (71.3%)	36 (52.9%)	0.03
JAK-STAT	*SOCS1, STAT3, STAT6*	41 (27.7%)	21 (26.3%)	20 (29.4%)	0.71
Immune escape	*B2M, CD70, CD58, CD83, CIITA*	64 (43.2%)	28 (35.0%)	36 (52.9%)	0.03
Apoptosis/cell cycle	*BCL2, FAS, PIM1, BTG1, CCND3*	93 (62.8%)	50 (62.5%)	43 (63.2%)	0.99
B cell development and differentiation	*MEF2B, IRF8, BCL6, PRDM1, EBF1, ETS1, IKZF3*	74 (50.0%)	39 (48.8%)	35 (51.5%)	0.87

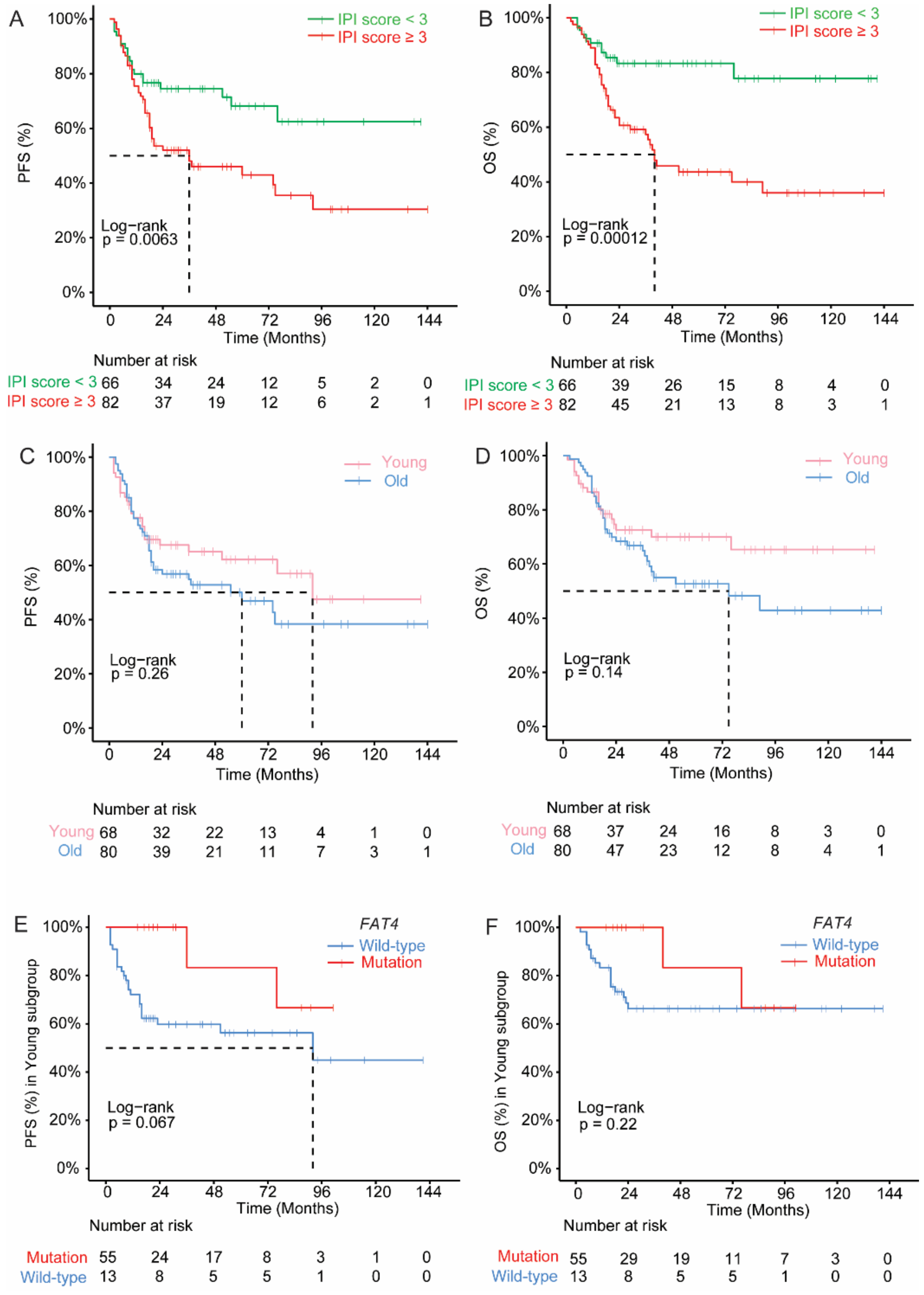

Supplementary Figure S1. The prognostic impact of IPI score, age, and *FAT4* mutation

The KM curves of PFS and OS in the entire cohort were analyzed based on IPI scores (A-B) and age at the diagnosis (C-D). In the sub-cohort of young (≤ 60 years) DLBCL patients, the prognostic impact of *FAT4* mutation on PFS (E) and OS (F) are shown by the KM curves, both of which are not statistically significant.

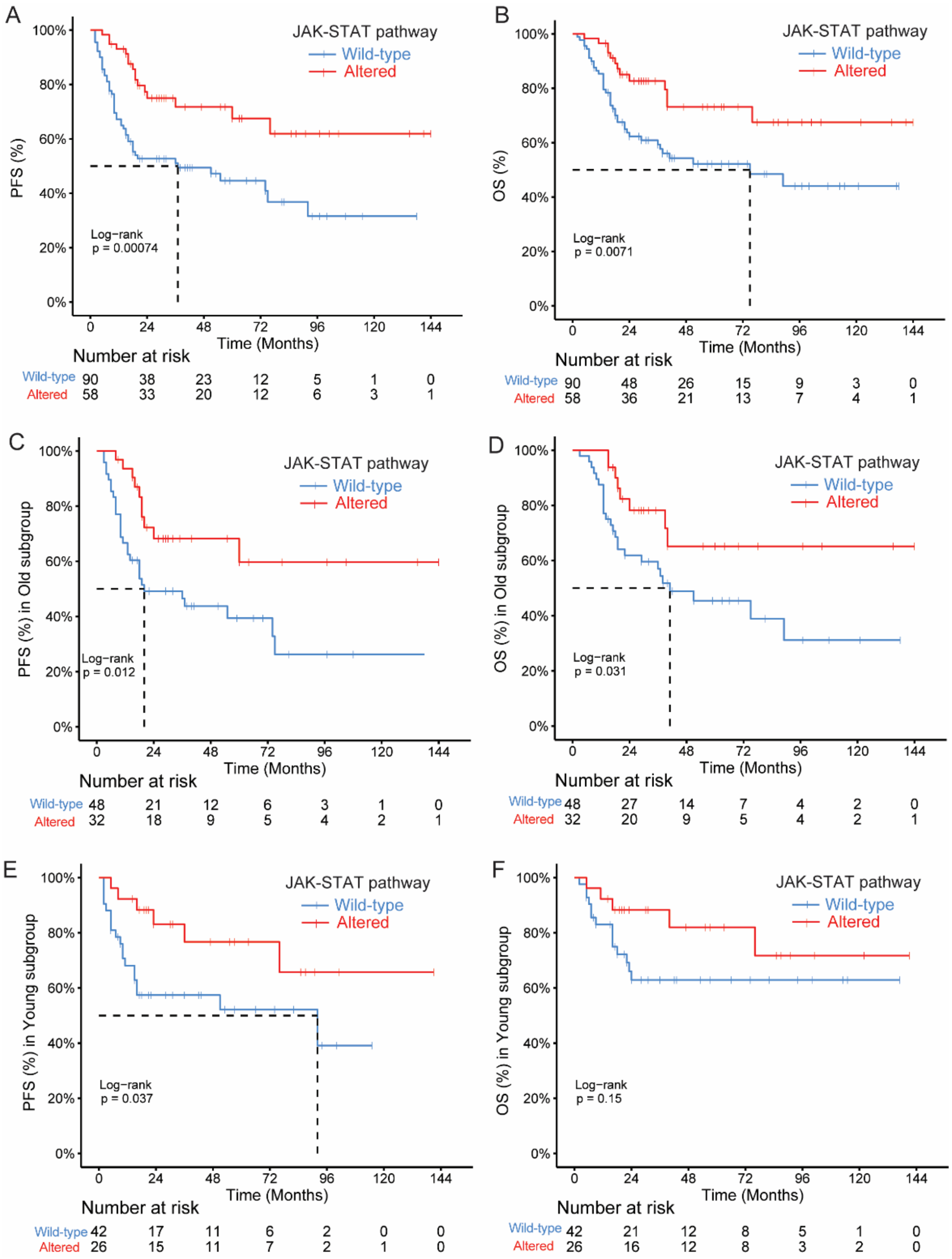

Supplementary Figure S2. JAK-STAT pathway alterations are associated with better survival.

The patients with altered JAK-STAT pathways (based on *SOCS1*, *STAT6*, and *FAT4* mutations) are associated with significantly longer PFS (A) and OS (B) in the entire cohort as well as in the old subgroup (C-D). The prognostic impact of the JAK-STAT pathway on PFS (E) is significant in the young subgroup, but not on OS analysis (F).
